# *Fusarium* Mycotoxins in Swiss Wheat: A Survey of Growers’ Samples between 2007 and 2014 Shows Strong Year and Minor Geographic Effects

**DOI:** 10.3390/toxins9080246

**Published:** 2017-08-09

**Authors:** Susanne Vogelgsang, Tomke Musa, Irene Bänziger, Andreas Kägi, Thomas D. Bucheli, Felix E. Wettstein, Matias Pasquali, Hans-Rudolf Forrer

**Affiliations:** 1 Agroscope, Reckenholzstrasse 191, 8046 Zurich, Switzerland; tomke.musa@agroscope.admin.ch (T.M.); irene.baenziger@agroscope.admin.ch (I.B.); andreas.kaegi@agroscope.admin.ch (A.K.); thomas.bucheli@agroscope.admin.ch (T.D.B.); felix.wettstein@agroscope.admin.ch (F.E.W.); hans-rudolf.forrer@agroscope.admin.ch (H.R.F.); 2 Department of Food, Environmental and Nutritional Sciences, University of Milan, Via Mangiagalli 25, 20133 Milano, Italy; matias.pasquali@unimi.it

**Keywords:** mycotoxin survey, wheat, *Fusarium*, trichothecene, zearalenone, food safety, Switzerland

## Abstract

To assess the occurrence of *Fusarium* toxins in wheat in Switzerland, an eight-year survey was conducted by analysing a total of 686 harvest samples from growers using LC-MS/MS. Between 2007 and 2010, 527 samples were obtained from 17 cantons. Between 2011 and 2014, 159 samples were collected from the canton Berne. The most frequent toxins detected were deoxynivalenol (DON), zearalenone (ZEA) and nivalenol (NIV). The overall mean DON content in all samples was 607 µg/kg, and 11% exceeded the European limit for unprocessed cereals for foodstuffs (1250 µg/kg). For ZEA (mean 39 µg/kg), 7% exceeded the respective limit (100 µg/kg), and the mean content of NIV (no limit established) was 15 µg/kg. Between the years, the ratio of mycotoxin-contaminated samples ranged between 52% and 98% for DON, 9% and 43% for ZEA and 0% and 49% for NIV. The yearly mean contents varied substantially between 68 and 1310 µg/kg for DON, 5 and 56 µg/kg for ZEA and 6 and 29 µg/kg for NIV. The geographic origin showed a significant effect on DON and ZEA contamination, but was inconsistent between the years. This study has shown that the majority of Swiss-produced wheat is, in terms of *Fusarium* toxins, fit for human consumption and feed purposes. Nevertheless, depending on the year, high toxin contents can be expected, an issue that growers, cereal collection centres and the food industry have to deal with to ensure food and feed safety.

## 1. Introduction

Small-grain cereals are commonly infected by various species from the fungal genus *Fusarium*, leading to Fusarium head blight (FHB) as one of the world most noxious cereal diseases [1]. Apart from severe yield losses, infected cereals are commonly contaminated with harmful mycotoxins, threatening human and animal health and, thus, rendering the harvested products inadequate for commercial uses. In the upper Midwest regions of the United States, the direct and secondary economic impacts of FHB epidemics between 1998 and 2000 in wheat and barley were estimated at $US 2.7 billion, accounting for 55% of total losses over that period [2,3].

From the 300 to 400 secondary metabolites recognised as mycotoxins [3], 150 belong to the group of trichothecenes, cyclic sesquiterpenoids with a 12,13-epoxide ring [4]. Trichothecenes, most commonly, but not exclusively, produced by *Fusarium* species, inhibit eukaryotic protein synthesis at the ribosomal level [5], and ingestion of contaminated grains may cause intestinal irritation in mammals, feed refusal in livestock, vomiting, skin dermatitis and immunological problems [6,7]. Trichothecenes have been classified into four groups [8], but type-A and type-B are the most prevalent trichothecenes occurring widely in cereals [9]. In Europe, *F. sporotrichioides* and *F. langsethiae* are the main producers of type-A trichothecenes, including T-2 and HT-2 toxins, diacetoxyscirpenol (DAS), monoacetoxyscirpenol (MAS), neosolaniol (NEO) and fusarenon-X (FUS-X) [10,11,12,13]. The type-B trichothecenes, such as deoxynivalenol (DON) and the co-contaminants 3- or 15-acetyl DON (3- or 15-Ac-DON), are produced predominantly by *F. graminearum* and *F. culmorum*, whereas nivalenol (NIV) is a common contaminant in cereals infected with *F. poae* [14,15,16]. Another mycotoxin frequently produced by *F. graminearum* is zearalenone (ZEA), a macrocyclic β-resorcyclic acid lactone, which possesses low cellular toxicity, but high estrogenic activity, commonly leading to hyperestrogenism and fertility problems [17].

In 2006, the European Commission set maximum limits for the *Fusarium* toxins DON, ZEA, as well as for fumonisins in cereals, maize and cereal/maize products for human consumption [18]. For T-2 and HT-2, recommendation levels for different cereal types have been established [19]. For NIV, no maximum limits or recommendations exist, despite the fact that its toxicity is more than ten-times higher than that of DON [5]. For feed materials and feed stuffs, guidance values for DON, ZEA and fumonisins exist [20].

A worldwide mycotoxin survey by BIOMIN, an animal health and nutrition company, in 2016, covering more than 16,000 agricultural commodity samples from 81 countries, showed that Europe is a high risk region with 71% DON incidence in cereals (wheat, barley, oats and rice) with an average content of 1023 µg/kg in the positive samples and a maximum of 37,640 µg/kg. For ZEA, the incidence in cereals was 36% with average and maximum contents of 78 and 3757 µg/kg, respectively [21].

Recent mycotoxin cereal surveys in European countries have shown that the species and mycotoxin patterns vary tremendously, depending on the geographic area, the host species, cropping factors and the climate in the respective sampling years [22]. In most countries, DON and NIV, as well as ZEA were predominant. However, in other environments, T-2 and HT-2 or even fumonisins were the most commonly detected mycotoxins. Furthermore, the majority of the studies cited above were performed for a short time period, spanning from two to a maximum of four years. To our knowledge, the only surveys conducted over a longer term (six to ten years) took place in Belgium [23], Norway [24], the U.K. [25] and Finland [26].

It is well known that the *Fusarium* species complex and the resulting mycotoxin contamination are highly dependent on the climatic factors shortly before, particularly during and sometimes after cereal anthesis [27,28,29,30]. Countries with very heterogeneous topographies face an even higher challenge to elucidate the mycotoxin risk in cereal production. Switzerland comprises 12 different climatic regions [31,32], due to the following circumstances: Firstly, the Alps act as a climatic barrier between northern and southern Switzerland. In the southern part, winters are considerably milder than in the northern part. Secondly, the complex mountain range of the Alps creates several additional climatic regions: In the inner-alpine valleys, such as the Grisons in the southeast of Switzerland, dry conditions prevail, since they are sheltered against precipitation from both the north, as well as the south. While the average amount of precipitation is close to 2000 mm per year along the northern foothills of the Alps, in the Alps and in southern Switzerland, regional amounts in the Grisons range between 600 and 700 mm per year. In the lowland north of the Alps, the amount is close to 1000 and 1500 mm per year. Thirdly, another special feature of the Swiss climate is the “Foehn” wind that crosses the Alps, creating explicitly mild and dry conditions on the leeward side. The southerly Foehn blowing from the south toward the north is often associated with high wind speeds in the north and high amounts of precipitation in the south.

Forecasting systems, particular those developed to predict mycotoxins in cereals, are powerful tools that help growers to prevent exceeding maximum limits and to avoid unnecessary fungicide applications [33,34,35,36]. The Swiss forecasting system FusaProg for DON in wheat [34], employing plot-specific cropping factors, growth stage and local weather data, was developed during the early 2000s. It is available to cereal growers since 2007 and has been validated with various samples from multi-year on-farm experiments [37].

The main objective of this study was to evaluate the risk of mycotoxin contamination of winter wheat in Switzerland over an extended time span. For this, growers’ samples from various cantons and climatic regions together with information on a number of cropping factors were collected. Based on measured mycotoxin contents, correlations between different mycotoxins were verified and it was assessed whether the sampling years and/or the geographic origin had an effect on the mycotoxin contamination. Another aim was to investigate whether the risk of DON contamination estimated by FusaProg correlates with measured DON contents, even by using samples from numerous cropping systems and geographic areas over several years. Finally, the contamination rate and the ratio of samples exceeding the maximum limits were determined.

## 2. Results

### 2.1. Sample Size and Origin

**Figure 1:**
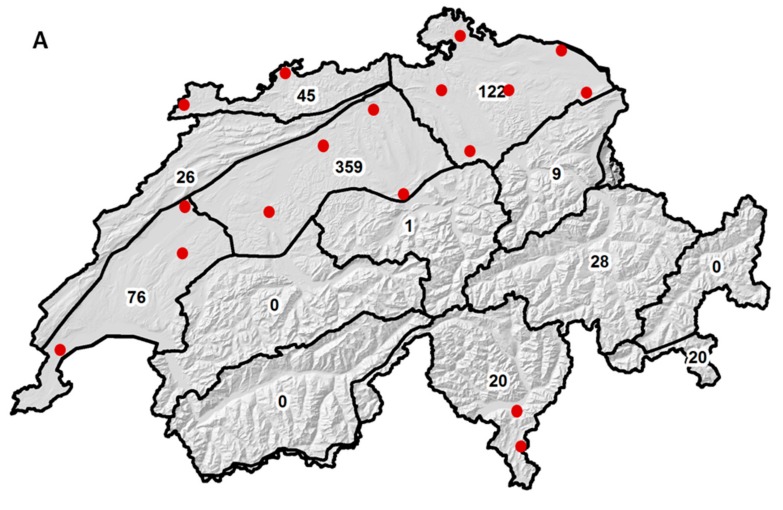

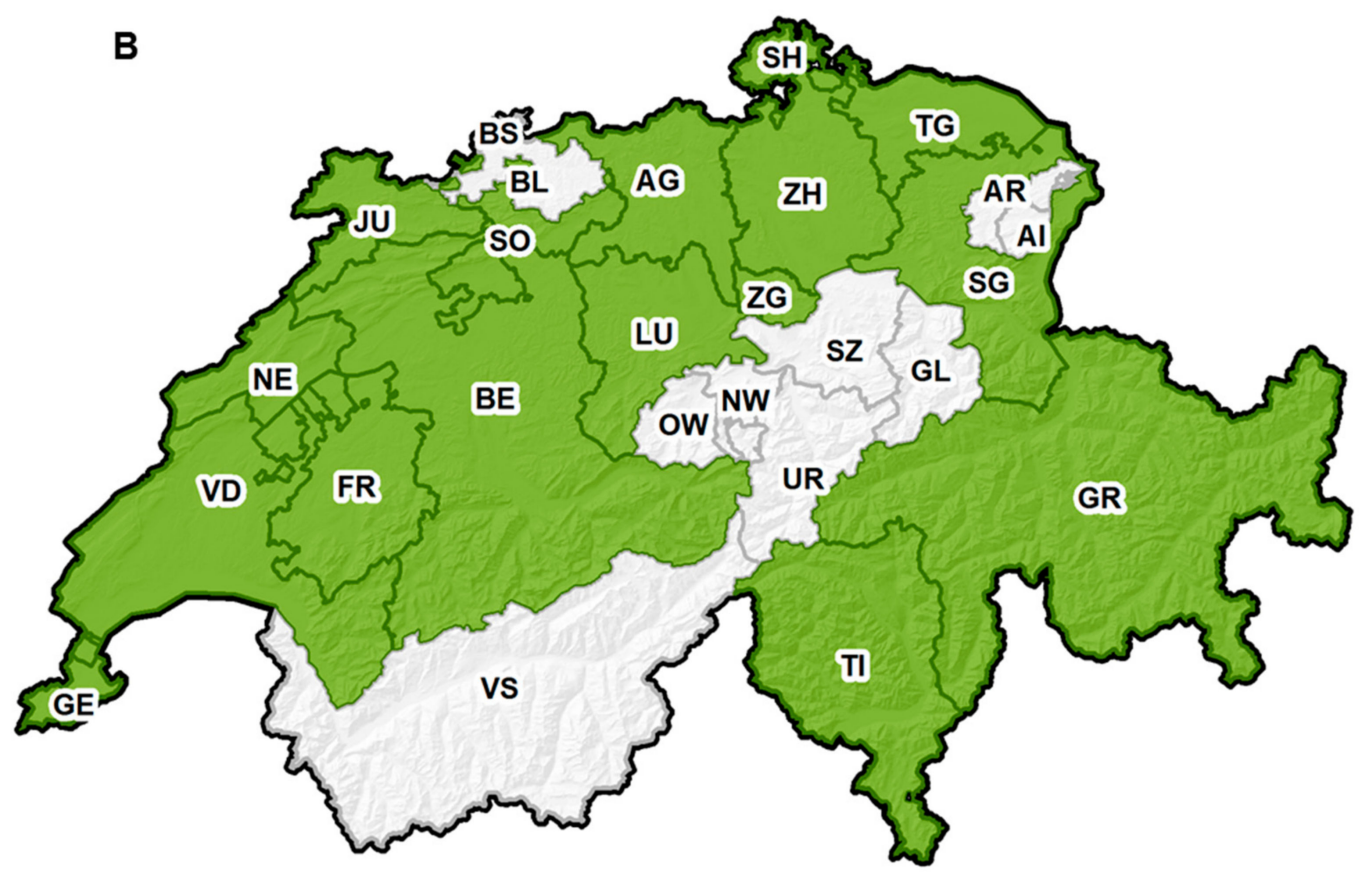
Swiss maps of climate regions and cantons. (**A**) Climate regions of Switzerland. Numbers in climate regions represent the number of wheat samples obtained. The 359 samples from the Central plateau include samples from both the Swiss-wide (2007–2010) and the canton Berne (2011–2014) monitoring. Corresponding names of the climate regions are described in the main text. Red circles indicate the location of the 17 weather stations used for the FusaProg (Swiss forecasting system for deoxynivalenol in wheat) calculation of the “Main Infection and Sporulation Periods”. No weather stations were used from the regions Western Jura, Northern and Central Grisons and Central Alpine northern slope, as samples were not received in all years. (**B**) Cantons of Switzerland. In the Swiss-wide monitoring, wheat samples were obtained from cantons marked in green. Unlabelled areas adjacent to cantons are small-sized enclaves of neighbouring cantons. Full names of abbreviated cantons from which samples were received are mentioned in the main text. The canton Schwyz (SZ) was not marked, as only one sample was obtained. Full names of the remaining cantons: Swiss federal states-cantons (http://swiss-government-politics.all-about-switzerland.info/swiss-federal-states-cantons.html). Map background: Hillshade and Cantonal Boundaries ©BFS/Federal Office of Topography, swisstopo, Swiss Climate Regions ©Federal Office of Meteorology and Climatology, MeteoSwiss.

In total, 686 wheat grain samples were analysed between 2007 and 2014. In the four years of the Swiss = wide monitoring, the sample numbers were 119 (year 2007), 129 (2008), 59 (2009) and 220 (2010), resulting in a total sample size of 527. In the years of the cantonal monitoring in Berne (total sample size of 159), the sample numbers were 36 (year 2011), 45 (2012), 45 (2013) and 33 (2014). Out of the 12 different climate regions of Switzerland, the samples were obtained from nine climate regions, including the Central plateau (359 samples), the North-eastern plateau (122), the Western plateau (76), the Eastern Jura (45), the Northern and Central Grisons (28), the Western Jura (26), the Alpine south side (20), the Eastern Alpine northern slope (9) and the Central Alpine northern slope (1) (Figure 1A). No samples were obtained from the climate regions Western Alpine northern slope, Valais and Engadine.

In terms of cantons, the samples between 2007 and 2010 originated from 17 different ones (Figure 1B), including Berne (85 samples), Aargau (53), Zürich (53), Jura (49), Zug (40), Thurgau (34), Neuchâtel (28), Vaud (27), Grisons/Graubünden (26), Luzern (24), Schaffhausen (23), Ticino (20), Solothurn (19), Fribourg (16), St. Gallen (16), Geneva (13) and Schwyz (1).

### 2.2. Detected Mycotoxins

Of the nine mycotoxins analysed from 686 harvest samples of wheat (see Section 5.3. in Materials and Methods), the most frequent toxins detected were DON, ZEA and NIV. Only a few samples were contaminated with 3- or 15-Ac-DON (20 samples), T-2 (11 samples), HT-2 (eight samples) or with NEO, DAS and FUS-X (one sample each), and hence, no statistics were performed with these toxin data.

### 2.3. Ratio of Samples Contaminated with DON, ZEA and NIV

The overall ratio of wheat grain samples between 2007 and 2014 with contents above the LOD were 80% for DON, 32% for ZEA and 21% for NIV. The ranges of DON, ZEA and NIV contents were between <LOD and 10,600, <LOD and 3070 and <LOD and 470 µg/kg, respectively, whereas the average contents of DON, ZEA and NIV were 607, 39 and 15 µg/kg, respectively. The overall ratio of mycotoxin-contaminated samples (% >LOD), mycotoxin means, the standard error of the mean, medians, 90% percentiles and maximum contents from the Swiss-wide monitoring (2007–2010), as well as from the monitoring in the canton of Berne (2011–2014) are shown in Table 1 and Table 2, respectively.

### 2.4. Correlation between the Mycotoxins DON, ZEA and NIV

Over all eight monitoring years, a highly significant (*p* < 0.001) correlation was observed between DON and ZEA, as well as between DON and NIV with r = +0.471 and r = +0.349, respectively. In contrast, the correlation between ZEA and NIV was not significant (*p* = 0.127, r = +0.058). However, the correlations varied substantially between the different monitoring years, ranging from r = +0.523–+0.916 between DON and ZEA, from r = +0.189–+0.604 between DON and NIV and from r = −0.034–+0.640 between ZEA and NIV (Table 3). Similarly, the correlation analysis based on samples where all three toxins were above the respective LOQs showed a significant (*p* = 0.013) correlation between DON and ZEA (r = +0.357) and a highly significant correlation (*p* < 0.001) between DON and NIV (r = +0.600); the correlation between ZEA and NIV was not significant (*p* = 0.670, r = +0.063).

### 2.5. Weather Data, Effect of the Year on Mycotoxin Contents and Estimation of the DON Risk through FusaProg

**Figure 2:**
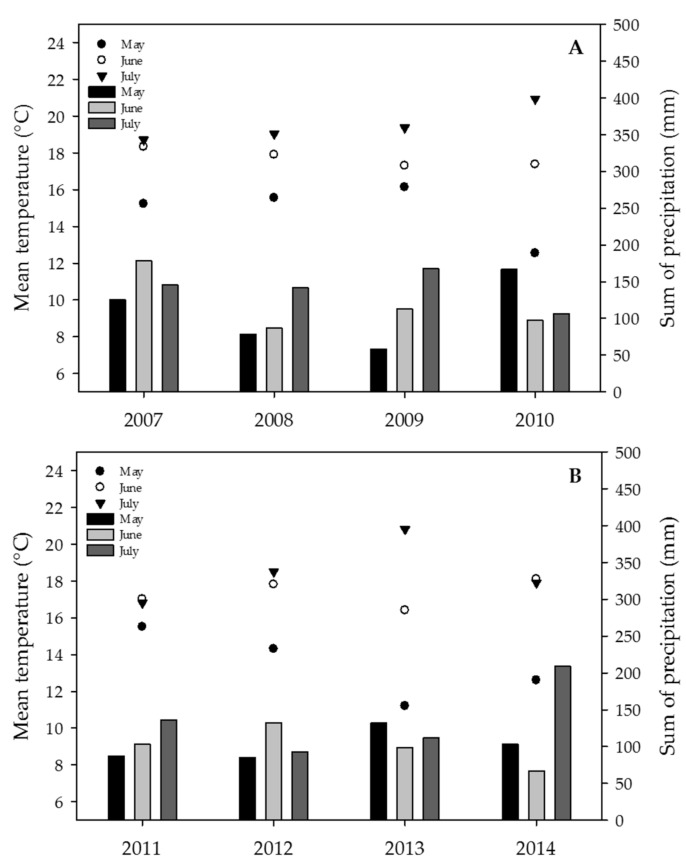
Weather conditions in May, June and July during the wheat mycotoxin monitoring between 2007 and 2014. (**A**) Swiss-wide monitoring (2007–2010); (**B**) monitoring in the canton Berne (2011–2014). Mean temperature (scatter symbols) and sum of precipitation (bars).

Depending on the year and the climate region, the mean temperature for the months of May–July ranged between 11 and 17 °C in May, between 16 and 20 °C in June and between 17 and 24 °C in July. A large variation in the monthly sum of precipitation was observed ranging from as low as 22 mm up to a maximum of 343 mm (Figure 2A,B) (data for different climate regions in Tables S1 and S2).

Pooled over all years in the Swiss-wide monitoring, large differences in average mean temperatures and precipitation from May to July were observed: the Eastern Jura, the North-eastern plateau and the Central plateau showed the lowest mean temperature (16, 17, 17 °C, respectively), whereas the Alpine south region clearly displayed the highest mean temperature (20 °C). With respect to precipitation, dry conditions prevailed more in the Western plateau and Eastern Jura (87 and 93 mm, respectively), while the Central plateau, the North-eastern plateau and the Alpine south regions experienced clearly more precipitation (127, 136 and 186 mm), respectively (Supplementary Table S1).

Between the monitoring years and throughout all climate regions, the estimated average onset of anthesis ranged between 21 May 2012 and 5 June 2013, whereas the end of anthesis ranged between 8 June 2008 and 25 June 2013.

**Figure 3:**
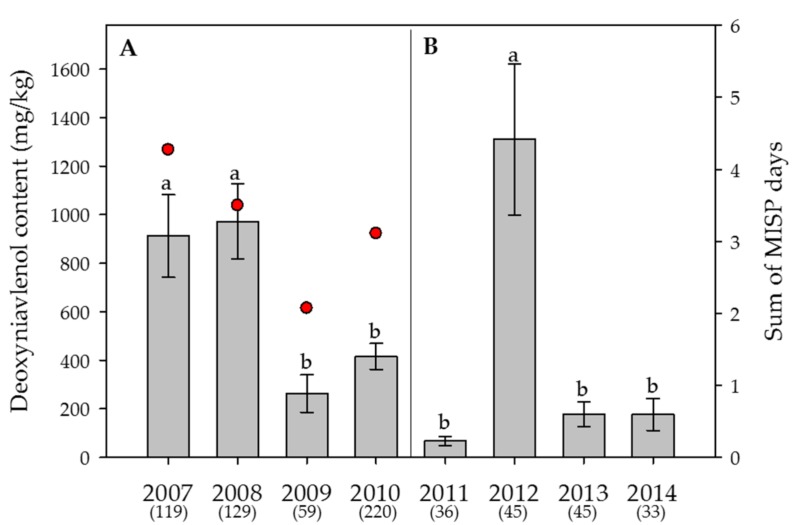
**Effect of the year** on the average content of **deoxynivalenol** in wheat. (**A**) Swiss-wide monitoring (2007–2010); (**B**) monitoring in the canton Berne (2011–2014). Numbers in parentheses indicate the number of samples. Error bars represent the standard error of the means. For each monitoring set, values followed by the same letters are not statistically different (α = 0.05). The red circles in (A) indicate the sum of the “Main Infection and Sporulation Period” (MISP) days (days with a high infection risk by *Fusarium graminearum*), averaged over 17 weather stations, calculated by the Swiss forecasting system FusaProg for the periods of anthesis for the years 2007 to 2010. No MISP calculations were done for the monitoring between 2011 and 2014.

**Figure 4:**
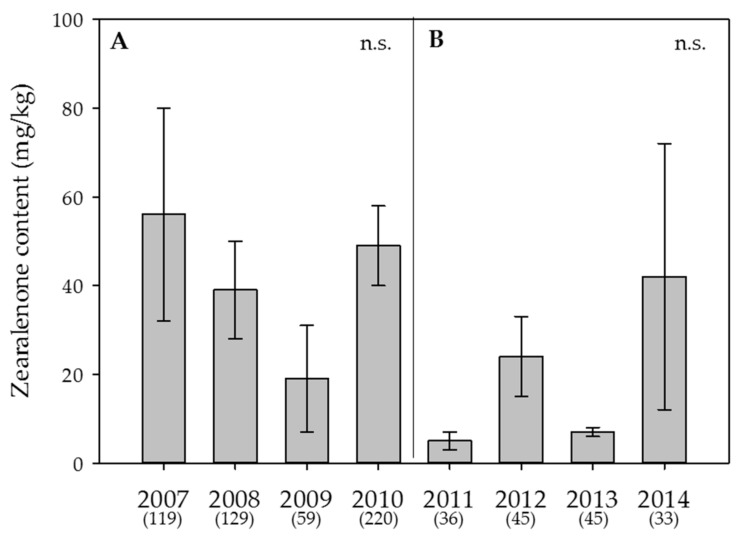
Effect of the year on the average content of **zearalenone** in wheat. (**A**) Swiss-wide monitoring (2007–2010); (**B**) monitoring in the canton Berne (2011–2014). Numbers in parentheses indicate the number of samples. Error bars represent the standard error of the means. n.s. = not significant (α = 0.05).

**Figure 5:**
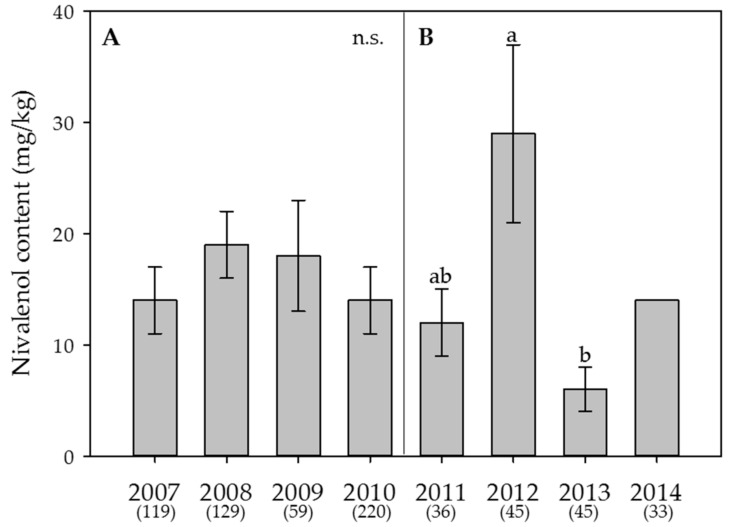
Effect of the year on the average content of **nivalenol** in wheat. (**A**) Swiss-wide monitoring (2007–2010); (**B**) monitoring in the canton Berne (2011–2014). For 2014, no statistical analyses were performed since the content in that year was always below the limit of detection. Numbers in parentheses indicate the number of samples. Error bars represent the standard error of the means. For each monitoring set, values followed by the same letters are not statistically different (α = 0.05); n.s. = not significant (α = 0.05).

Within the individual years 2007 and 2014, the ratio of samples where DON was detected ranged between close to half to almost 100%, i.e., in 88%, 93%, 75%, 80%, 53%, 98%, 56% and 52% of all samples, respectively. ZEA was detected in fewer samples, namely in 43%, 39%, 17%, 34%, 19%, 42%, 9% and 15% of all samples, respectively. NIV was found in 20%, 34%, 19%, 11%, 39%, 49%, 9% and 0% of all samples, respectively. The yearly averages ranged between 68 and 1,310 µg/kg for DON, 5 and 56 µg/kg for ZEA and 6 and 29 µg/kg for NIV (Figure 3, Figure 4 and Figure 5).

In the Swiss-wide monitoring, the year had a highly significant effect on the average content of DON (*p* < 0.001) (Figure 3A), but not on ZEA (*p* > 0.05) (Figure 4A) or NIV (*p* > 0.05) (Figure 5A). Despite the significant effect of the year on DON, only 4.3% (eta square value of η^2^ = 0.043) of the overall variation was explained by the year. For DON, the years with the highest contents were 2007 and 2008. In the canton Berne monitoring, the year had a significant effect on DON (*p* < 0.001; η^2^ = 0.18) (Figure 3B) and NIV (*p* = 0.004, η^2+^ = 0.082) (Figure 5B), but not on ZEA (*p* > 0.05) (Figure 4B). Here, 2012 was the year with the highest DON contents (Figure 3B).

For the years with the significantly highest DON contents (2007, 2008 and 2012), the temperature and precipitation did not necessarily reflect these measurements, and only trends were observed. Nevertheless, for 2007, the mean temperature in July was not as warm as those in 2009 and 2010. For 2008, more precipitation and cooler temperatures were recorded in July compared with those observed in 2010 (Figure 2A, Table S1). Within the canton Berne monitoring, the mean precipitation for the Central and Western plateau in June of 2012 was clearly the highest compared with the precipitation in June of the years 2011, 2013 and 2014 (Figure 2B, Table S2).

Within the Swiss-wide monitoring, the years with the highest average DON contents (2007 and 2008) also showed the highest sum of the “Main Infection and Sporulation Period” (MISP) days, calculated by FusaProg (72 and 59 days, respectively), during the periods of anthesis. Accordingly, the year with the lowest average DON content (2009), was also reflected in the lowest number of MISPs (35 days) (Figure 3A). The risk assessment of FusaProg being below or above a threshold level of 1000 µg/kg was correct in 88% of the 298 wheat samples, where no fungicide treatments were indicated in the respective questionnaires. Out of the 12% of wrong estimations, 8% were cases where FusaProg underestimated the DON content, whereas 4% were cases where the model overestimated the DON content (data not shown).

### 2.6. Effect of Sample Origin on Mycotoxin Contents between 2007 and 2010

Pooled across all years, the highest average contents of DON were observed in the Eastern Jura (1214 µg/kg, *n* = 45) and the Eastern Alpine northern slope (1211 µg/kg, *n* = 9), whereas the lowest average content was found in the Northern and Central Grisons (203 µg/kg, *n* = 28) (Table 4). Despite the significant effect (*p* = 0.014), only 2.6% of the variation of DON contents was explained by the climate region, and the all-pairwise multiple comparison (Games-Howell) did not detect significant differences between the means. No effect of the climate region was observed on the contents of ZEA or NIV.

With respect to the cantons, the highest and the lowest average contents of DON were observed from samples in the canton of Jura (1314 µg/kg, *n* = 49) and Fribourg (159 µg/kg, *n* = 16), respectively. The contents in samples from Fribourg and Grison/Graubünden were significantly lower (*p* < 0.001) than those in samples from the canton Zug. For ZEA, samples from several cantons showed average contents below the LOD (Fribourg (*n* = 16), Geneva (*n* = 13), Neuchâtel (*n* = 28)), and the highest average was detected in Zug (204 µg/kg, *n* = 40). Although there was a significant effect of the cantonal origin on the ZEA contents (*p* < 0.001), the all-pairwise multiple comparison did not detect significant differences between the means. For NIV, the cantonal origin had no significant effect, and most cantons provided samples with low average contents. Samples from Geneva had the highest average (50 µg/kg, *n* = 13), but the effect was not significant. The canton Schwyz was not considered for any mycotoxin comparison since only one sample was obtained.

### 2.7. Ratio of Samples Exceeding Legal Limits for Food or Guidance Values for Feed

Throughout all years, 77 (11%) or 48 (7%) samples contained a DON or ZEA content above the European maximum level for unprocessed cereals for human consumption, 1250 and 100 µg/kg [18], respectively. Levels per year varied between 0% and 27% for DON and between 0% and 12% for ZEA (Table 5). With respect to feed material [20], only five (0.7%) or two (0.3%) samples contained a DON or ZEA content above the guidance values of 8000 and 2000 µg/kg, respectively.

## 3. Discussion

In the current study, the risk of *Fusarium* mycotoxin contaminations of winter wheat in Switzerland was evaluated by analysing growers’ harvest samples from various regions over several years.

### 3.1. Detected Mycotoxins and Correlations

The predominant mycotoxins were DON, followed by ZEA and NIV, whereas other *Fusarium* mycotoxins were only sporadically detected. This finding is most probably due to the presence of three of the most frequent *Fusarium* species in Europe, *F. graminearum*, *F. poae* and *F. culmorum*, e.g., [14,38,39,40], although the latter seems to be decreasing during the last decades [41,42]. Even on a global level, such as in Argentina, Australia, Brazil and Canada, these toxins are considered to be the most frequent ones in small-grain cereals [43,44,45,46].

In all monitoring years, the Pearson correlation coefficient analyses revealed a highly significant correlation between DON and ZEA. This result underlines the capacity of *F. graminearum* to produce both toxins within a single isolate, e.g., [47]. The correlations between DON and NIV were not as consistent, but were still highly significant in five out of the eight monitoring years. This might be explained by either the co-occurrence of *F. graminearum*, *F. poae* and/or *F. culmorum* in the analysed wheat grain samples and/or by the presence of both DON and NIV chemotypes of *F. graminearum* [48]. In fact, a recent large-scale study has demonstrated the co-occurrence of 3-Ac-DON, 15-Ac-DON and NIV chemotypes of *F. graminearum* in various European countries [49]. The relationship between ZEA and NIV was substantially lower, and 2012 was the only year with a highly significant correlation. This result might be explained by distinct environmental requirements for a given *Fusarium* species or strain to produce different mycotoxins [50], as well as by the potential interactions between environment, disease control measures and *Fusarium* species/isolates [51,52,53,54]. In addition, interactions between *Fusarium* species and other co-occurring pathogenic fungal genera are known to influence the production of *Fusarium* mycotoxins, as has been shown in an in vitro study by Sass et al. [55]. In the current monitoring study with numerous different environmental and cropping conditions, these interactions cannot be investigated; hence, experiments under a controlled environment with different species, strains and a range of abiotic factors would be needed to better understand these mycotoxin correlations.

### 3.2. Effect of the Year and the Sample Origin

The monitoring year demonstrated a substantial effect on both the DON contamination rate and the average content. Nevertheless, the effect size in the Swiss-wide monitoring, as described by the partial eta square value, was rather low, compared with that of the canton Berne monitoring. This result might be due to the extremely variable topography in Switzerland within climate regions, as well as to cropping factors, partially overriding the year effects. Nevertheless, a mycotoxin survey of swiss granum, an association of cereal cultivation organisations, underlines the year as a crucial factor for DON contamination: Yearly mean DON contents of bread wheat sampled at cereal collection centres from 2007 to 2014 (992 samples in total) varied between 121 and 480 µg/kg. Despite the smaller range of contents, there was a strong positive correlation between the yearly swiss granum data and the results of the current study (r^2^ = 0.818) (Table S3, Figure S1). The substantially lower DON contents of the swiss granum survey can be explained by wheat grain cleaning steps before the sampling and analyses and the observation that Swiss bread wheat compared with fodder wheat varieties is often less susceptible to *F. graminearum* infection and subsequent toxin accumulation. Although not significant, the ZEA content also varied considerably between the different years. For NIV, the effect of the year was significant in the canton Berne monitoring, but the effect size was also rather small. The overall small eta square values suggest that other factors strongly influence *Fusarium* infections and mycotoxin contamination. Nevertheless, the prevailing temperature and moisture immediately before and during anthesis have a strong effect, not only on the maturation of the perithecia (requirement of 20–25 °C and RH ≥ 85%), but also on the discharge of ascospores of *F. graminearum* (optimum 21 °C and RH = 100%) [56,57], leading to infection and subsequent contamination with DON and ZEA and, depending on the chemotype, also with NIV. Thus, the high average DON contents in the years 2007 and 2012 can be partially explained by the weather variables in June, where the majority of the wheat crops were in the growth stages of anthesis to early post-anthesis. For example, in 2007, the month of June was not as warm as in 2009 and 2010; and within the canton Berne monitoring, the mean precipitation for the Central and Western plateau in June of 2012 was clearly the highest compared with the precipitation in June of 2011, 2013 and 2014. The high average DON content in the year 2008, a year with an early average anthesis period, cannot be explained by favourable temperature or precipitation during the second half of May and the first week of June. However, in 2008, the sum of FusaProg calculated MISP days during the estimated anthesis period was clearly higher compared with that in 2009, the year with the lowest average DON content in the Swiss-wide monitoring. Hence, this finding suggests that even a few days with temperature and moisture favourable for *F. graminearum* during anthesis can be crucial for infection and ultimately decisive for mycotoxin production. Another reason for the year-to-year variability might be the presence of various other FHB-causing *Fusarium* species within wheat samples and even within a single kernel. As demonstrated in a single-isolate versus co-inoculation study with different temperature and wetness conditions, infections by more than one *Fusarium* species are considered to lead to competitive interactions resulting in reduced fungal DNA on the one hand, but could lead to an overall strong increase of DON and NIV on the other [58]. Despite contrasting results with respect to mixed inoculations, ranging between decrease, increase or similar levels compared with single-isolate inoculation, depending on the fungal species and environmental conditions [53], it would be important to determine the fungal amount of the dominant species in the wheat samples from the current study to better understand the yearly variability of the main toxins detected. Although *F. graminearum* infections most often lead to contamination of both DON and ZEA, the yearly average ZEA contents were highly variable and, in the canton Berne monitoring, did not always follow the patterns of DON. This could be explained by a number of studies that reported that post-anthesis rainfall, especially in combination with delayed wet harvests, significantly increased ZEA contents, whereas ZEA contents remained low in the absence of moisture late in the season, even under severe FHB incidence [59,60]. In addition, field inoculations with *F. culmorum* followed by weekly sampling until harvest showed that DON was detected in higher contents and in earlier growth stages, while ZEA was found later and in smaller amounts [61,62]. These results support the hypothesis that environmental conditions during certain growth stages do not have equivalent effects on the accumulation of all mycotoxins from a single *Fusarium* species. Remarkably, in 2014, which was the year with the highest ZEA contents in the canton Berne monitoring, the month of July with more than 200 mm precipitation was by far the wettest pre-harvest month from all years of the current study. The yearly contents of NIV were even less correlated with those of DON, which could be due to the co-occurrence of *F. graminearum*, *F. poae* and/or *F. culmorum* as described above or by the shift of chemotype composition that was shown to be affected by climatic conditions [63]. In addition, it might also be possible that NIV contamination occurs irrespective of climate conditions as has been demonstrated in a two-year wheat monitoring in Italy [12]. In another study between 2001 and 2004, where wheat samples from Hungary, Ireland and the United Kingdom were analysed, significant correlations were found between relative humidity variables and DON, as well as between temperature and relative humidity variables and NIV for time windows that started after anthesis [64]. However, due to the high variability in the observed relationships, the authors suggested that no single environmental variable is sufficient for the prediction of mycotoxin contamination.

Certainly, the best approach to predict *Fusarium* mycotoxin contamination in small-grain cereals, especially for DON, is to combine weather data with cropping factors known to influence the infection risk. This has been demonstrated in a modelling study by Gourdain et al. [65], as well as in a two-year field study analysing the effect of environmental, topographic and agricultural management factors on DON and ZEA in more than 300 wheat samples in Germany [66]. In fact, this combination is successfully employed in the Swiss forecasting system FusaProg, and for 298 wheat samples of the current dataset (no fungicide treatments), the FusaProg calculations resulted in 88% correct risk estimates.

Averaged over the monitoring years 2007 to 2010, the mean contents of DON originating from different climate regions were lowest in the Northern and Central Grisons and highest in the Eastern Jura, although this factor described only 2.6% of the overall variability. Indeed, the wide range observed cannot be explained by the respective weather conditions from May to July as in the Northern and Central Grisons, the temperature represented the exact average of all climate regions (17.4 °C), and the mean sum of precipitation (118 mm) was only slightly lower than the average (122 mm) of all climate regions. Likewise, the Eastern Jura experienced even less precipitation (93 mm) than most other climate regions, and its mean temperature was rather cool at 16.3 °C. Furthermore, the climate region had no effect on the average contents of ZEA or NIV. The fact that samples originating from certain cantons contained higher or lower average DON contents has to be interpreted with even greater caution. Due to the very heterogeneous topography in Switzerland, with numerous valleys and influences from lakes and rivers, as well as the Alps, a given canton cannot be characterised by one single weather pattern. Moreover, we assume that agricultural practices as mentioned above have a large effect on the occurrence of FHB, particularly for the main species *F. graminearum* and its main toxins DON and ZEA. Hence, a forthcoming detailed multivariate analysis of various cropping factors indicated in the questionnaires will help to elucidate the reasons for elevated contents, not only for DON and ZEA, but also for NIV.

### 3.3. Ratio of Samples Exceeding the Legal Limits for Food or Guidance Values for Feed

The ratio of samples exceeding maximum limits for DON and ZEA in unprocessed wheat for human consumption ranged between 0% and 27% (swiss granum survey between 0% and 6%) and between 0% and 12%, respectively. The partially high values of the current study could be quite alarming in terms of food safety and should be addressed by appropriate cropping strategies to reduce the risk. Nevertheless, the cleaning and sorting procedures in the grain collection centres remove small and shrunken kernels, which frequently contain the highest amounts of DON produced by *F. graminearum*. In contrast, infections of *F. poae* do not lead to this kind of kernel symptom [16]; hence, NIV contamination cannot be reduced by these cleaning procedures. In comparison, the ratio of samples exceeding guidance values for DON and ZEA for feed material was less than 1%. With respect to the toxicological relevance, the composition of feedstuff has to be considered. In Switzerland for example, the ratio of wheat grains in swine feed might comprise up to 40% (personal communication by Claude Chaubert, Agroscope), depending on the current price in a given year. Moreover, grain and/or silage maize, which are also commonly used as feed ingredients, are frequently even more contaminated with *Fusarium* toxins and a wider range of toxic metabolites due to the broader species spectrum compared with wheat [67,68]. Furthermore, a comprehensive review by Grenier and Oswald [69], analysing 112 reports on the co-occurrence of different mycotoxins from various fungal genera, revealed that most of the included studies showed a synergistic or additive interaction on the adverse effects of animal performance. However, the results on other parameters, such as biochemical compounds, indicated for the same mycotoxin combination different types of interactions, from synergistic to antagonistic. Nonetheless, the authors concluded that exposure to a co-contaminated food/feed would result in a greater risk to human and animal health.

## 4. Conclusions

The current study, examining 686 wheat samples throughout eight years, has shown that Swiss wheat is frequently contaminated by *Fusarium* mycotoxins, in particular by DON, ZEA and NIV. With the current factors analysed, the greatest variability was observed by the monitoring year, especially for DON, whereas the geographic origin showed only marginal effects. Although it is known that infection by the most prevalent species *F. graminearum* is influenced by selected cropping factors, e.g., [70], present efforts focus on analysing the effects of previous crops, tillage, wheat variety, cropping system, fertiliser and plant protection products on the incidence and the fungal DNA amounts of several FHB-causing species and the resulting mycotoxin accumulation. Despite the fact that currently, only DON and ZEA in wheat are regulated by maximum limits for food and guidance values for feed, these upcoming analyses will also aim at elucidating potential factors that could limit the risk of infection by NIV producing *Fusarium* species, which might represent an underestimated threat for human and animal health.

## 5. Materials and Methods

### 5.1. Sampling

During the growing seasons of 2007 to 2010, Swiss growers from all wheat growing areas were contacted each year and asked to participate in this survey by providing a harvest sample of wheat grains. Subsequently, between 2011 and 2014, further wheat samples were obtained from growers of the canton Berne, representative for the Swiss Central and Western plateau for wheat production, who participated in a cantonal program of soil protection (“Kantonales Förderprogramm Boden”/“Programme cantonal de promotion des sols”) [71]. In the written request for a harvest sample, instructions were given on how to take the sample, consisting of removal of ten samples (approximately 1 kg each) from different locations directly from the combine harvester, thorough mixing and taking a pooled mixture of 1 kg. Furthermore, a questionnaire was included to obtain information on agricultural practices, as well as on plot location, sowing and harvest dates, the beginning of anthesis and grain yield. Growers sent these samples in plastic bags overnight along with agronomic data pertaining to each field sample. For all processing steps, uncleaned grain samples were used.

### 5.2. Preparation of Subsamples

The moisture content of grains was determined with a grain analysis computer (Model GAC^®^ 2100 AGRI, DICKEY-john Corporation, Effretikon, Switzerland). If needed, grains were placed in open trays with a metal mesh at the bottom and dried with forced air at 32 °C for 2 to 3 days at a minimum of 1000 m^3^/h/ton (drying and heating chamber, Model FED 400, Binder GmbH, Tuttlingen, Germany) to a maximum of 14% grain moisture. After the drying process, the grain samples were stored at 10 °C until analyses to avoid any degeneration of the samples. To ensure a random distribution of different grain fractions, samples were further processed using a grain divider (sample splitter RT6.5, Retsch GmbH, Haan, Germany). A sub-sample of 10 g was finely ground with a sample mill (Model 1093, Tecator, Cyclotec 1093, IG AG, Zurich, Switzerland, mesh size 1 mm), and the resulting flour was stored at −20 °C until analysis.

### 5.3. Quantification of Toxins

For determination and quantification of mycotoxins, liquid chromatography tandem mass spectrometry (LC-MS/MS) using a 1200 L system (Varian Inc., Walnut Creek, CA, USA) was performed. Details are described in Forrer et al. [72], and certified wheat reference flours were used (Trilogy, Washington, MO, USA). The analytes were DON, ZEA, NIV, acetyl-deoxynivalenol (Ac-DON: sum of 3-Ac-DON and 15-Ac-DON), NEO, DAS, HT-2 and T-2 toxin and FUS-X. Each analyte was detected with two transitions (qualifier and quantifier) in multiple reaction monitoring (MRM). Analyte identification was confirmed using chromatographic retention time, correct mass of the mother ion, correct mass of the two daughter ions and agreement of the ratio of qualifier to quantifier with the calibration (±10%). For quantification, the method of matrix matched calibration was implemented to correct for possible ion suppression. Recoveries for low (0.5 mg/kg) and high (2 mg/kg) spiked blank samples (*n* = 4) were between 86–126 and 78–107%, respectively. Method precision was in the range of 2 and 12%, whereas instrument precision was between 2% and 10%. The toxin measurements were conducted over a period of several years; hence, due to differences in the sensitivity of the LC-MS/MS instrument, detection and quantification limits varied from one sample run to the other. For samples obtained from the harvests between 2007 and 2013, the limits of detection (LOD, in μg/kg) for DON ranged between 5 and 26, for ZEA between 1 and 9 and for NIV between 3 and 20. The limits of quantification (LOQ, in μg/kg) for DON ranged between 18 and 32, for ZEA between 5 and 19 and for NIV between 10 and 37. For the samples of 2014, a second LC-MS/MS instrument of the same type with partially higher detection and quantification limits had to be used (DON: LOD 13–20, LOQ 43–65; ZEA: LOD 9–10, LOQ 31–32; NIV: LOD 5–27, LOQ 16–91). For samples in which no toxin was detected or detected, but not quantified, and to allow statistical analyses, half of the respective LOD or LOQ was used, respectively. Although the other analytes, Ac-DON, NEO, DAS, HT-2/T-2 toxin and FUS-X, were detected only sporadically (less than 3% of all samples), LODs/LOQs (including those from the year 2014) ranged between 3-13/9-45, 1-5/5-16, 1-2/2-8, 6-22/20-73, 1-7/3-23 and 3-13/10-, respectively.

### 5.4. Estimation of the DON Risk through the Forecasting System FusaProg

Based on plot-specific cropping factors, growth stage and local weather data, FusaProg can predict the risk of a *F. graminearum* infection and estimate the subsequent DON contamination in wheat. Since less than 30% of the growers indicated the onset of wheat anthesis, observations from the Cantonal Plant Protection Services and from Agroscope cereal experiments were used to estimate the overall onset and duration of wheat anthesis (from decimal code (DC) growth stages 61–69 [73]) for each individual monitoring year, spanning a range for early maturing varieties in lower altitudes until late maturing varieties in higher altitudes. Based on the prevailing weather data from 17 weather stations in the regions where samples were obtained, the weather model in FusaProg calculates the number of days with a low (factor 0), medium (factor 0.33) or a high risk (factor 1.0) of *F. graminearum* between May and August [34]. The resulting sum divided by the number of weather stations represents the “Main Infection and Sporulation Period” (MISP) days for each year and was plotted together with the yearly average DON content measured. These calculations were only performed for the Swiss-wide monitoring since in the canton Berne monitoring, the number of samples was very low, and for one out of the four years, reliable observations for the onset and duration of wheat anthesis were missing. For samples where no fungicide treatment was indicated in the respective questionnaire (*n* = 298), the ratio of correct FusaProg DON estimations, being either below or above a given threshold of 1000 µg/kg, was calculated.

### 5.5. Statistical Analyses

Descriptive statistics on mycotoxins and contents were performed including the percent of samples with mycotoxin contents that were above the LOD, as well as mean, standard error of means, median, 90th percentiles and maximum contents. Two-tailed Pearson correlations (significance level at 0.01) between DON, ZEA and NIV were computed both for individual monitoring years and pooled across all years. The correlations were also performed with samples where the contents of all three toxins were above the respective LOQs. Since the MISP based on FusaProg was a single value for each year, no correlations were conducted between the MISP and the measured DON content in the obtained samples. A univariate general linear model (GLM) was used to analyse the effect of the year and the origin (climate region and the canton) on the average toxin contents. To estimate the effect size, that is the contribution of a factor to the overall variability, the partial eta square value η^2^ was calculated [74]. When the overall effect of the relevant factor in the GLM was significant and to evaluate differences between means (α = 0.05), an all-pairwise multiple comparison procedure for uneven sample sizes according to Hochberg’s GT2 (Generalized Tukey 2) (in the case of equal variances) or according to Games–Howell (unequal variances, non-parametric) was performed [75]. The number of samples exceeding legal limits for food or guidance values for feed was calculated and expressed in percent. Analyses were performed using IBM SPSS Statistics for Windows, Version 24. Graphs were plotted using SigmaPlot, Version 13.0 (Systat Software, San Jose, CA, USA).

## Figures and Tables

**Table 1:** Contents of the *Fusarium* mycotoxins deoxynivalenol (DON), zearalenone (ZEA) and nivalenol (NIV) detected in Swiss wheat grain samples (17 cantons) between 2007 and 2010 (*n* = 527).

		Mycotoxin Content (µg/kg) ^a^				
	% >LOD	Mean	SEM ^b^	Median	90th Percentile	Maximum
DON	84	647	60	160	3686	10,600
ZEA	34	45	9	3	190	3070
NIV	19	15	2	4	78	470

^a^ Based on the following limit of detection (LOD) and limit of quantification (LOQ) ranges (in µg/kg): DON: LOD 5–26, LOQ 18–65; ZEA: LOD 1–10, LOQ 5–32; NIV: LOD 3–27, LOQ 10–91. For samples with contents below the LOD or the LOQ, LOD/2 and LOQ/2 were used for calculation, respectively. ^b^ SEM = standard error of the mean.

**Table 2:** Contents of the *Fusarium* mycotoxins deoxynivalenol (DON), zearalenone (ZEA) and nivalenol (NIV) detected in wheat grain samples from the canton Berne between 2011 and 2014 (*n* = 159).

		Mycotoxin Content (µg/kg) ^a^				
	% >LOD	Mean	SEM ^b^	Median	90th Percentile	Maximum
DON	66	473	99	100	2510	9880
ZEA	22	18	7	5	47	1000
NIV	25	16	3	3	75	290

^a,b^ See Table 1.

**Table 3:** Pearson correlation coefficients between the *Fusarium* mycotoxin contents of deoxynivalenol (DON), zearalenone (ZEA) and nivalenol (NIV) detected in wheat including data from the Swiss-wide *Fusarium* toxin monitoring between 2007 and 2010 and the monitoring in the canton Berne between 2011 and 2014. *n* = number of samples.

Year (*n*)		ZEA	NIV
2007 (119)	DON	+0.575 **	+0.588 **
	ZEA	-	+0.172 ^n.s.^
2008 (129)	DON	+0.573 **	+0.391 **
	ZEA	-	+0.211 *
2009 (59)	DON	+0.916 **	+0.217 ^n.s.^
	ZEA	-	+0.067 ^n.s.^
2010 (220)	DON	+0.523 **	+0.189 **
	ZEA	-	-0.034 ^n.s.^
2011 (36)	DON	+0.616 **	+0.527 **
	ZEA	-	+0.162 ^n.s.^
2012 (45)	DON	+0.767 **	+0.604 **
	ZEA	-	+0.640 **
2013 (45)	DON	+0.796 **	+0.306 *
	ZEA	-	+0.208 ^n.s.^
2014 (33)	DON	+0.880 **	n.a.
	ZEA	-	n.a.

** Significant at *p* < 0.001; * significant at *p* < 0.01; n.s., not significant; n.a., not available since the contents in that year were always below the limit of detection.

**Table 4:** Effect of the sample origin (climate region) on the average content (µg/kg) of deoxynivalenol (DON), zearalenone (ZEA) and nivalenol (NIV) in wheat from the Swiss-wide *Fusarium* toxin monitoring between 2007 and 2010. *n* = the number of samples. Numbers in parentheses represent the standard error of the mean.

Climate Region ^a^	*N*	DON ^b^	ZEA (n.s.)	NIV (n.s.)
Eastern Jura	45	1214 (203)	27 (30)	16 (6)
Western Jura	26	876 (267)	19 (40)	13 (8)
N-E plateau	122	404 (123)	30 (18)	14 (4)
Central plateau	201	717 (96)	81 (14)	14 (3)
Western plateau	75	598 (157)	8 (23)	30 (4)
E Alpine N-slope	9	1211 (455)	100 (67)	12 (13)
N-C Grisons	28	203 (258)	11 (38)	4 (7)
Alpine south side	20	441 (305)	12 (45)	4 (9)

^a^ N-E = North-eastern, E = Eastern, N-slope = Northern slope, C = Central, N-C = Northern Central. N = number of wheat samples. ^b^ Despite the overall significant effect (*p* = 0.014), the all-pairwise multiple comparison (Games–Howell) did not detect significant differences between the means. n.s. = not significant (α = 0.05).

**Table 5:** Percentage of samples exceeding the European legal limits ^a^ of deoxynivalenol (DON: 1250 µg/kg) and zearalenone (ZEA: 100 µg/kg) in unprocessed cereals intended for human consumption, including data from the Swiss-wide *Fusarium* toxin monitoring between 2007 and 2010 and the monitoring in the canton Berne between 2011 and 2014. *n* = number of samples.

Year	*n*	% Samples DON >1250 µg/kg	% Samples ZEA >100 µg/kg
2007	119	16.0	9.2
2008	129	19.4	10.1
2009	59	1.7	1.7
2010	220	7.7	12.3
2011	36	0.0	0.0
2012	45	26.7	6.7
2013	45	4.4	0.0
2014	33	3.0	6.1
**Overall**	**686**	**11.2**	**7.0**

^a^ http://eur-lex.europa.eu/legal-content/EN/TXT/PDF/?uri=CELEX:32006R1881&from=en.

## Supplementary Materials

The following are available online at www.mdpi.com/2072-6651/9/8/246/s1: Table S1: Regional distribution of wheat samples and weather conditions in May, June and July during the Swiss-wide *Fusarium* toxin monitoring years 2007–2010. Table S2: Weather conditions in the Central and Western plateau (canton Berne) in May, June and July during the *Fusarium* toxin monitoring years 2011–2014. Table S3: Correlation of the deoxynivalenol (DON) contents between the swiss granum survey and the current study. Figure S1: Results from the Swiss granum analysis program for deoxynivalenol in bread wheat 2007–2014.
