# Correction: S. Vogelgsang et al. *Fusarium* Mycotoxins in Swiss Wheat: A Survey of Growers’ Samples between 2007 and 2014 Shows Strong Year and Minor Geographic Effects. *Toxins* 2017, *9*, 246

**DOI:** 10.3390/toxins9110377

**Published:** 2017-11-21

**Authors:** Susanne Vogelgsang, Tomke Musa, Irene Bänziger, Andreas Kägi, Thomas D. Bucheli, Felix E. Wettstein, Matias Pasquali, Hans-Rudolf Forrer

**Affiliations:** 1 Agroscope, Reckenholzstrasse 191, 8046 Zurich, Switzerland; tomke.musa@agroscope.admin.ch (T.M.); irene.baenziger@agroscope.admin.ch (I.B.); andreas.kaegi@agroscope.admin.ch (A.K.); thomas.bucheli@agroscope.admin.ch (T.D.B.); felix.wettstein@agroscope.admin.ch (F.E.W.); hans-rudolf.forrer@agroscope.admin.ch (H.-R.F.); 2 Department of Food, Environmental and Nutritional Sciences, University of Milan, Via Mangiagalli 25, 20133 Milano, Italy; matias.pasquali@unimi.it

The authors wish to correct Figures 3–5 in this paper [1]. In all three figures, the y axis stated as a unit “milligram/kg” (mg/kg) instead of “microgram/kg” (µg/kg).

Replace

**Figure 3:**
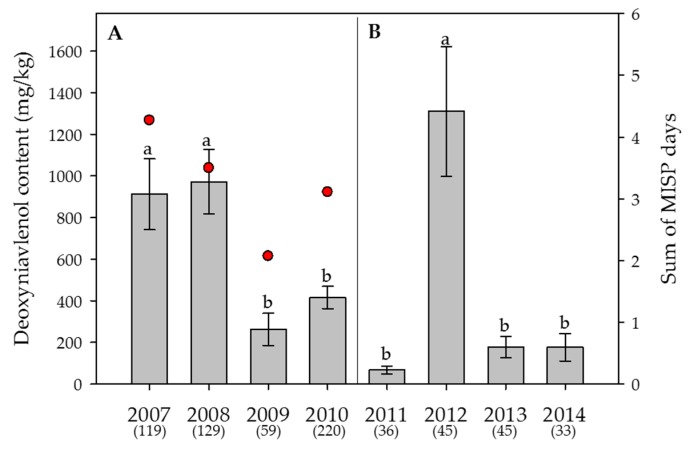
**Effect of the year** on the average content of **deoxynivalenol** in wheat. (**A**) Swiss-wide monitoring (2007–2010); (**B**) monitoring in the canton Berne (2011–2014). Numbers in parentheses indicate the number of samples. Error bars represent the standard error of the means. For each monitoring set, values followed by the same letters are not statistically different (α = 0.05). The red circles in (**A**) indicate the sum of the “Main Infection and Sporulation Period” (MISP) days (days with a high infection risk by *Fusarium graminearum*), averaged over 17 weather stations, calculated by the Swiss forecasting system FusaProg for the periods of anthesis for the years 2007 to 2010. No MISP calculations were done for the monitoring between 2011 and 2014.

with

**Figure 3:**
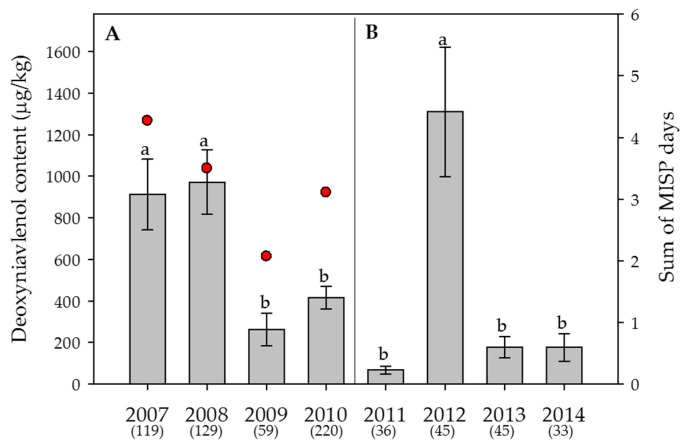
**Effect of the year** on the average content of **deoxynivalenol** in wheat. (**A**) Swiss-wide monitoring (2007–2010); (**B**) monitoring in the canton Berne (2011–2014). Numbers in parentheses indicate the number of samples. Error bars represent the standard error of the means. For each monitoring set, values followed by the same letters are not statistically different (α = 0.05). The red circles in (**A**) indicate the sum of the “Main Infection and Sporulation Period” (MISP) days (days with a high infection risk by *Fusarium graminearum*), averaged over 17 weather stations, calculated by the Swiss forecasting system FusaProg for the periods of anthesis for the years 2007 to 2010. No MISP calculations were done for the monitoring between 2011 and 2014.

Replace

**Figure 4:**
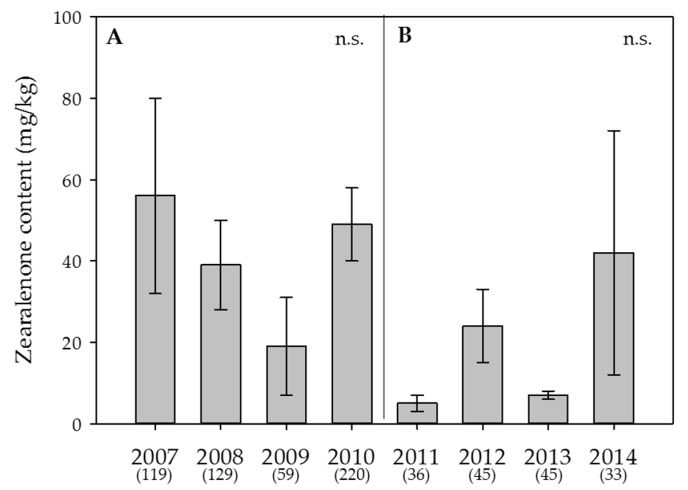
**Effect of the year on the average content of zearalenone in wheat.** (**A**) Swiss-wide monitoring (2007–2010); (**B**) monitoring in the canton Berne (2011–2014). Numbers in parentheses indicate the number of samples. Error bars represent the standard error of the means. n.s. = not significant (α = 0.05).

with

**Figure 4:**
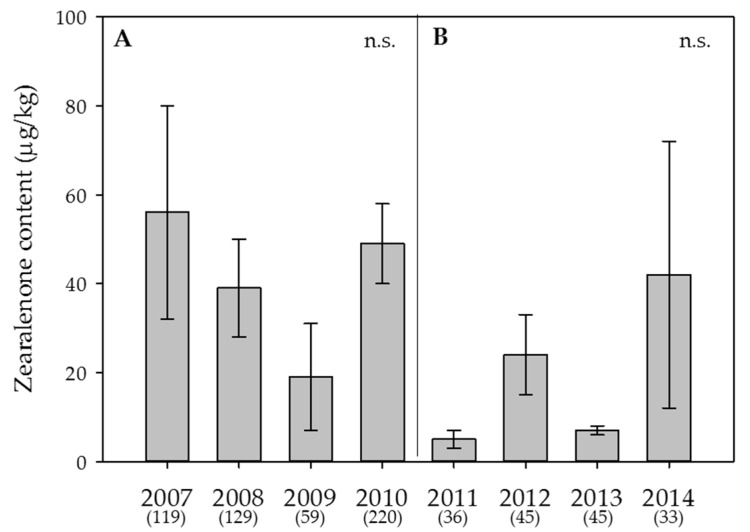
**Effect of the year on the average content of zearalenone in wheat.** (**A**) Swiss-wide monitoring (2007–2010); (**B**) monitoring in the canton Berne (2011–2014). Numbers in parentheses indicate the number of samples. Error bars represent the standard error of the means. n.s. = not significant (α = 0.05).

Replace

**Figure 5:**
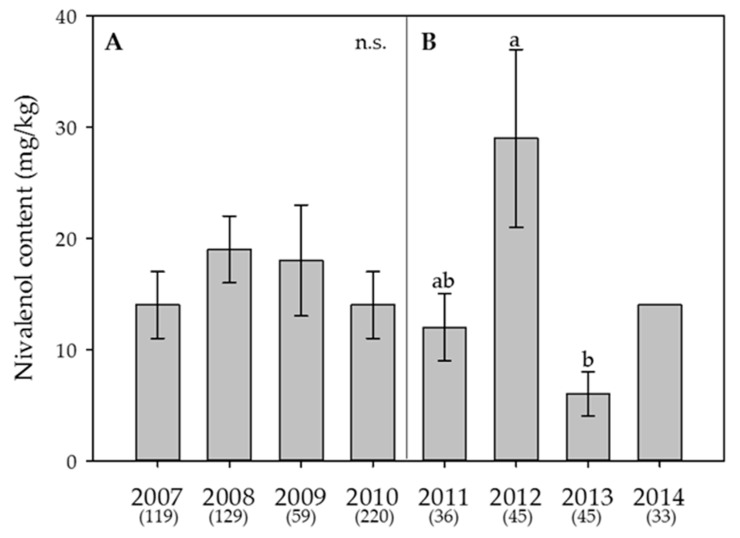
**Effect of the year on the average content of nivalenol in wheat.** (**A**) Swiss-wide monitoring (2007–2010); (**B**) monitoring in the canton Berne (2011–2014). For 2014, no statistical analyses were performed since the content in that year was always below the limit of detection. Numbers in parentheses indicate the number of samples. Error bars represent the standard error of the means. For each monitoring set, values followed by the same letters are not statistically different (α = 0.05); n.s. = not significant (α = 0.05).

with

**Figure 5:**
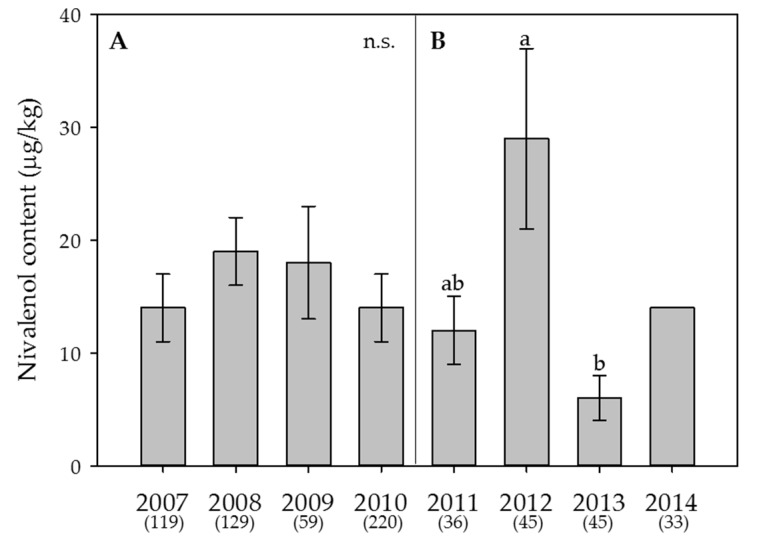
**Effect of the year on the average content of nivalenol in wheat.** (**A**) Swiss-wide monitoring (2007–2010); (**B**) monitoring in the canton Berne (2011–2014). For 2014, no statistical analyses were performed since the content in that year was always below the limit of detection. Numbers in parentheses indicate the number of samples. Error bars represent the standard error of the means. For each monitoring set, values followed by the same letters are not statistically different (α = 0.05); n.s. = not significant (α = 0.05).

The changes do not affect the scientific results. The manuscript will be updated and the original will remain online on the article webpage. We apologize for any inconvenience caused to our readers.

## References

[1] Vogelgsang S., Musa T., Bänziger I., Kägi A., Bucheli T.D., Wettstein F.E., Forrer H.R. (2017). *Fusarium* Mycotoxins in Swiss Wheat: A Survey of Growers’ Samples between 2007 and 2014 Shows Strong Year and Minor Geographic Effects. Toxins.

