# Supplementary material for: Fusarium Mycotoxins in Swiss Wheat: A Survey of Growers’ Samples between 2007 and 2014 Shows Strong Year and Minor Geographic Effects
Source: Toxins (Basel). 2017 Aug 9;9(8):246. doi: 10.3390/toxins9080246 (PMC5577580; doi:10.3390/toxins9080246)
Supplement: Supplementary file 1 [file toxins-09-00246-s001.zip › Supplementary_Tables_S1_S2.pdf]

Table S1: Regional distribution of wheat samples and weather conditions in May, June and July during the Swiss wide *Fusarium* toxin monitoring years **2007 to 2010**.

			Eastern Jura	N-E plateau	Central plateau	Western plateau	E Alpine N-slope	N-C Grisons	Alpine south
		N	45	122	201	76	9	28	20
**2007**	Mean temperature (°C)	May	14.6	14.8	14.5	14.6	15.5	15.2	17.4
		June	17.5	17.9	17.7	18.0	18.6	18.5	20.1
		July	17.6	17.9	17.7	18.2	18.9	18.7	22.0
	Sum of precipitation (mm)	May	123	150	178	134	88	73	129
		June	205	134	175	153	142	172	266
		July	107	163	194	153	156	146	98
**2008**	Mean temperature (°C)	May	15.0	15.3	15.2	15.0	16.8	-	16.0
		June	16.8	17.3	17.5	17.7	18.2	-	19.9
		July	18.3	18.2	18.6	19.0	18.7	-	21.4
	Sum of precipitation (mm)	May	55	37	33	59	22	-	263
		June	45	116	74	54	66	-	165
		July	99	147	127	107	186	-	186
**2009**	Mean temperature (°C)	May	14.6	15.5	15.4	15.9	-	16.9	18.5
		June	15.6	16.7	16.7	17.1	-	17.3	20.4
		July	18.0	18.7	18.7	19.4	-	19.5	21.9
	Sum of precipitation (mm)	May	56	100	77	40	-	19	56
		June	82	144	126	83	-	80	161
		July	89	156	178	92	-	146	343
**2010**	Mean temperature (°C)	May	11.3	11.7	12.1	12.6	12.5	12.5	15.1
		June	16.4	16.4	16.7	17.2	17.4	17.6	19.9
		July	20.1	19.7	20.1	21.5	20.5	20.6	24.1
	Sum of precipitation (mm)	May	127	167	140	89	136	147	360
		June	74	148	103	52	89	50	164
		July	56	173	117	23	180	153	44

N = number of wheat samples. N-E = North-eastern, E = Eastern, N-slope = Northern slope, C = Central, N-C = Northern central. “-“ = no samples obtained. Data from Western Jura and Central Alpine north slope are not included as samples were obtained from one year and one single sample, respectively.

Table S2: Weather conditions in the Central and Western plateau (canton Berne) in May, June and July during the *Fusarium* toxin monitoring years **2011 to 2014**.

Year (N)	Mean temperature (°C)			Sum of precipitation (mm)		
	May	June	July	May	June	July
2011 (36)	15.5	17.0	16.8	87	103	136
2012 (45)	14.3	17.8	18.5	85	132	93
2013 (45)	11.2	16.4	20.8	132	99	112
2014 (33)	12.6	18.1	17.9	103	67	209

N = number of wheat samples
