# Supplementary material for: Fusarium Mycotoxins in Swiss Wheat: A Survey of Growers’ Samples between 2007 and 2014 Shows Strong Year and Minor Geographic Effects
Source: Toxins (Basel). 2017 Aug 9;9(8):246. doi: 10.3390/toxins9080246 (PMC5577580; doi:10.3390/toxins9080246)
Supplement: Supplementary file 1 [file toxins-09-00246-s001.zip › Supplementary_Table_S3.pdf]

Table S3:

Results from the swiss granum analysis programme for deoxynivalenol (DON) in bread wheat 2007-2014

swiss granum

Schweizerische Branchenorganisation Getreide, Ölsaaten und Eiweisspflanzen
Organisation de la Branche Suisse des Céréales, Oléagineux et Protéagineux

swiss granum
Postfach 7957
3001 Bern
Tel. +41 (0)31 385 72 72
Fax +41 (0)31 385 72 75
info@swissgranum.ch
www.swissgranum.ch

Results from the swiss granum analysis programme for bread wheat (pre-sorted samples, second last cleaning step)

	2007	2008	2009	2010	2011	2012	2013	2014
**Number of analysed samples**	130	132	131	122	126	132	115	104
Samples with 0 mg/kg DON	48%	50%	63%	56%	79%	32%	62%	92%
n =	62	66	83	69	100	42	71	96
Samples with > 0 - 0.75 mg/kg DON	39%	43%	34%	34%	21%	49%	38%	8%
n =	51	57	45	41	26	65	44	8
Samples with > 0.75 – 1.25 mg/kg DON	11%	5%	2%	7%	0%	13%	0%	0%
n =	14	7	2	9	0	17	0	0
Samples with > 1.25 – 2 mg/kg DON	0%	0%	1%	1%	0%	5%	0%	0%
n =	0	0	1	1	0	6	0	0
Samples with > 2 mg/kg DON	2%	2%	0%	2%	0%	1%	0%	0%
n =	3	2	0	2	0	2	0	0

0: below the limit of detection (200 µg/kg)

Source: swiss granum and Agroscope
