# Supplementary material for: Fusarium Mycotoxins in Swiss Wheat: A Survey of Growers’ Samples between 2007 and 2014 Shows Strong Year and Minor Geographic Effects
Source: Toxins (Basel). 2017 Aug 9;9(8):246. doi: 10.3390/toxins9080246 (PMC5577580; doi:10.3390/toxins9080246)

Figure S1:

Correlation of the deoxynivalenol (DON) contents between the swiss granum survey and the current study

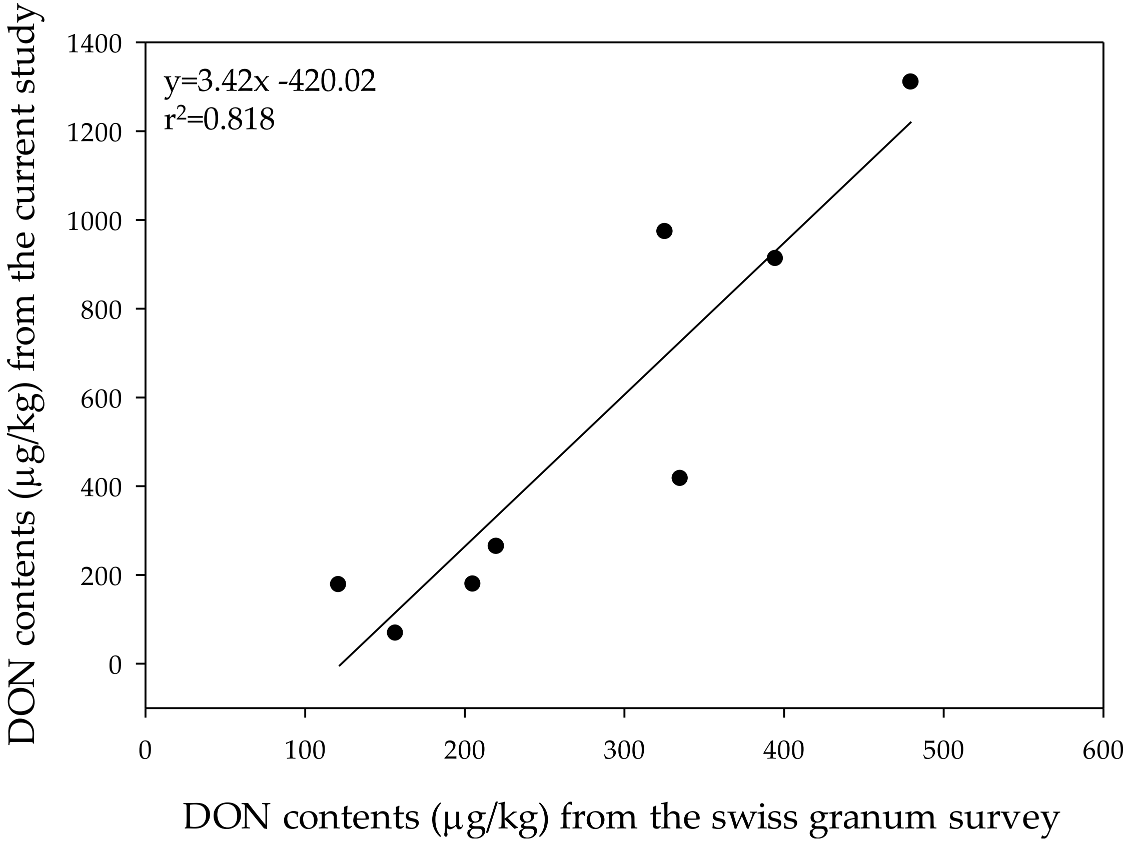

Supplement: Supplementary file 1 [file toxins-09-00246-s001.zip › Supplementary_Figure_S1.pdf]
